# Supplementary figures and images for: History of tuberculosis disease is associated with genetic regulatory variation in Peruvians
Source: PLoS Genet. 2024 Jun 13;20(6):e1011313. doi: 10.1371/journal.pgen.1011313 (PMC11208071; doi:10.1371/journal.pgen.1011313)

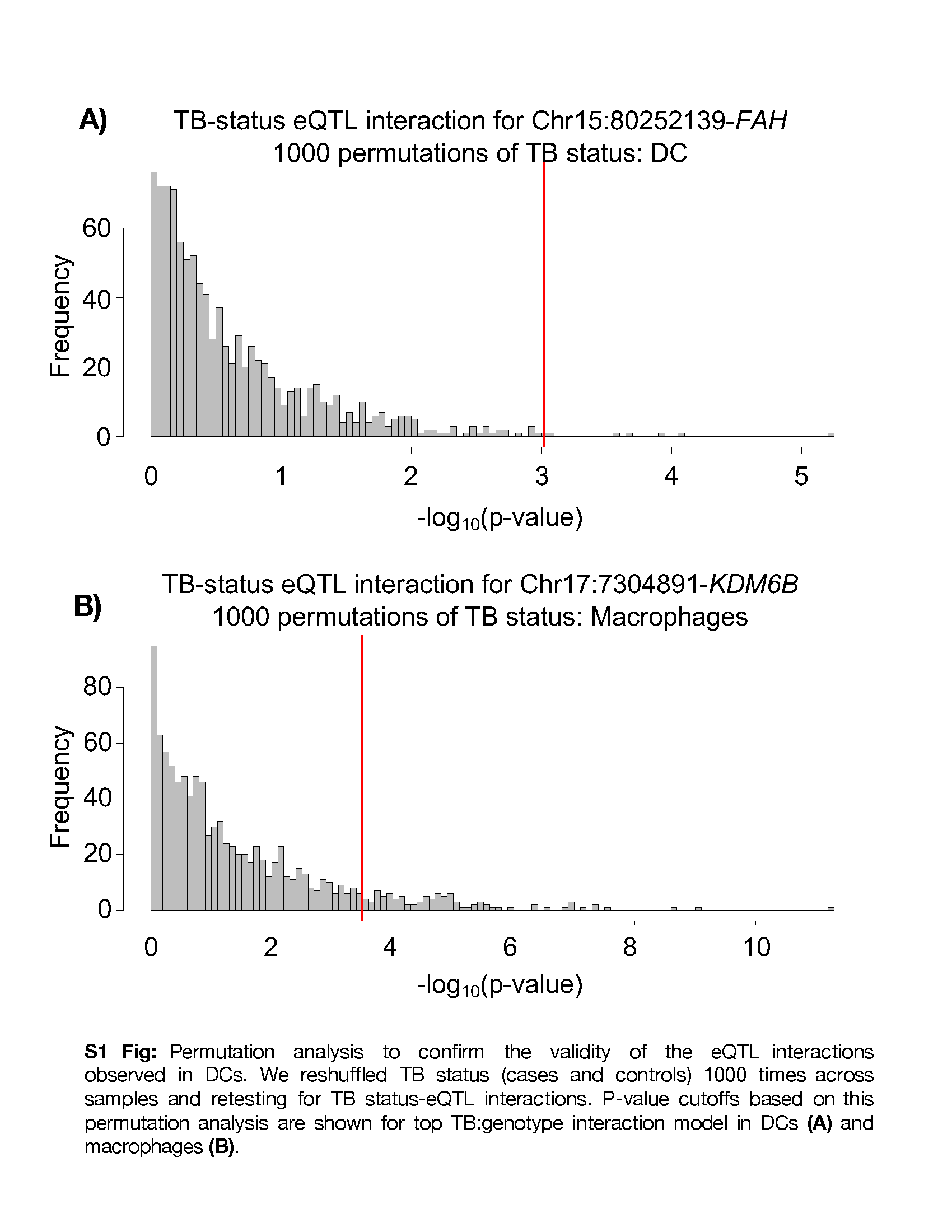

Supplement: S1 Fig — We reshuffled TB status (cases and controls) 1000 time across samples and retesting for TB status-eQTL interactions. P-value cutoffs based on this permutation analysis are shown for top TB:genotype interaction model in DCs (A) and macrophages (B). (TIFF) [file pgen.1011313.s001.tiff]
